# Identifying competitive strategies to improve the performance of hospitals in a competitive environment

**DOI:** 10.1186/s12913-017-2699-9

**Published:** 2017-11-21

**Authors:** Chuan-Hui Chang, Yu-Ching Chiao, Yafang Tsai

**Affiliations:** 1 Department of Business Administration, National Chung Hsing University, Taichung, Taiwan; 2 Central Division, National Health Insurance Administration , Ministry of Health and Welfare, Taichung, Taiwan; 3 Department of Health Policy and Management, Chung Shan Medical University, Taichung, Taiwan; 4 Department of Medical Management, Chung Shan Medical University Hospital, Taichung, Taiwan

**Keywords:** Competitive dynamics, National health insurance, Global budget system, Type of hospital action, Performance

## Abstract

**Background:**

This study is based on competitive dynamics theory, and discusses competitive actions (including their implementation requirements, strategic orientation, and action complexity) that influence hospitals’ performance, while also meeting the requirements of Taiwan’s “global budget” insurance payment policy.

**Methods:**

In order to investigate the possible actions of hospitals, the study was conducted in two stages. The first stage investigated the actions of hospitals from March 1 to May 31, 2009. Semi-structured questionnaires were used, which included in-depth interviews with senior supervisors of 10 medium- and large-scale hospitals in central Taiwan. This stage collected data related to the types of actions adopted by the hospitals in previous years. The second stage was based on the data collected from the first stage and on developed questionnaires, which were distributed from June 29 to November 1, 2009. The questionnaires were given to 20 superintendents, deputy superintendents, and supervisors responsible for the management of a hospital, and focused on medical centers and regional hospitals in central Taiwan in order to determine the types and number of competitive actions.

**Results:**

First, the strategic orientation of an action has a significantly positive influence on subjective performance. Second, action complexity has a significantly positive influence on the subjective and the objective performance of a hospital. Third, the implementation requirements of actions do not have a significantly positive impact on the subjective or the objective performance of a hospital.

**Conclusion:**

Managers facing a competitive healthcare environment should adopt competitive strategies to improve the performance of the hospital.

## Background

Subsequent to the establishment of the National Health Insurance (NHI) system, Taiwan’s National Health Insurance Administration (NHIA) faced financial pressure due to increasing medical expenses. In 2002, it implemented a healthcare insurance payment policy called the “global budget,” which replaced the existing “fee-for-service” policy. The purpose of the global budget policy was to control the expenses of the entire medical care reimbursement system. However, in order to secure a portion of the limited healthcare budget, the policy increased competition among hospitals within the same domain, prompting hospital managers to reform their operation models. These policy changes were successful in decelerating hospital revenues and expenditures [1]. Under these conditions, hospital managers face the challenge of taking appropriate competitive actions.

Therefore, this study explores the types of strategic orientation that need to be adopted and their effects on performance in hospitals in Taiwan that function within competitive environments. The research outcomes could assist in boosting the confidence of hospital managers. Previous studies on competitive strategies have generally used either a static analysis [2] or the Herfindahl–Hirschman Index (HHI) [1, 3–5]. This study is based on competitive dynamics theory, and explores the competitive actions (including their strategic orientation, action complexity, and implementation requirements) that influence hospitals’ performance, while meeting the requirements of Taiwan’s global budget insurance payment policy.

### Perspective of awareness-motivation-capability (AMC)

When organizations function in a market environment with competitive dynamics, they should adopt strategies based on the changes occurring in the market. For instance, organizations change their strategies after observing other competitors in the market. Chen et al. [6] extended the AMC theory, where responses (stimulations) to competitive actions are initiated only when rivals become aware of the competitive move and they possess the motivation and capability to respond. In contrast, when facing a strategic or challenging competitive move, the likelihood of initiating a response is minimal in the case of low capability [7]. The AMC perspective proposes that a firm will respond to a stimulation only when it is aware of the action and is motivated to respond [8].

### Characteristics of competitor actions

From an AMC perspective, before initiating an action or a response, an organization or a rival needs to become aware of the actions of the competitor, as well as the changes occurring in the organization’s external environment. Awareness is essentially consciousness, and motivation is a combination of psychological and cognitive phenomena [9]. A “strategic orientation” affects the likelihood of an attack on other competitors. Here, a rival should consider the “action complexity” and the “implementation requirements” before responding with the motivation necessary to achieve the expected result.

### Implementation requirement of an action

Implementation requirements refer to the effort that an initiator devotes to an action—an extreme promise index for an attack—because an organizational promise and implementation requirements are essential for coordinating a competitive action [10]. Therefore, implementation requirements describe the type of initiator and provide information on a competitive action promise [10]. In practice, there are multiple resources involved in the implementation requirements, including the economy, regulation, organization, psychology, and government precepts, all of which present the message on the type of action type for the attack [10]. Chen et al. [6] state that an attack with high implementation requirements reduces the number of competitive responses and increases their lag time. Most rivals are not willing to respond to a firm’s attack, and the speed of their responses is generally low [6], which results in better performance of the attacking firm. Thus, a hospital will exhibit better performance when it undertakes actions with a higher implementation requirement.

### Strategic orientation of action

In general, competitive action sends a specific message to the markets, which could be visible or implicit. Subsequently, other organizations will evaluate and manage this message in order to achieve success in the market [11]. Organization information processing theory examines the information flow inside and surrounding an organization in order to explain organizational behavior [12]. However, there are several types of competitive actions, with various messages, and each type is associated with a particular scope and cost. These strategic actions include competitive actions with significant investments in fixed assets, staff, and organizational structure change, or radical innovation to work around industry regulations [6], including equipment expansion, mergers and acquisitions, alliances, and introducing new products or services [13]. In contrast, tactical actions refer to the actions that do not have specific commitments to fixed assets, have less of an effect on the initiator in the long term [6], and have small and routine transformations that are mostly completed by lower-level managers using a minimum resource commitment. These actions can be managed by thoroughly revising current procedures and do not require serious structural adjustments [6], such as price cuts, advertisements, or gradual adjustments of products or services [13].

Broadly speaking, an organization’s response to a strategic action could be vague and uncertain. This can be attributed to managers undertaking the majority of the decisions, or organizational information not translating external information into internal concepts through information processing [11]. In addition, time and energy are required to deal with such information. In order to achieve better efficiency, managers initiate strategic actions. However, because the information is often considerably uncertain, in order to minimize risks, they may wait to obtain clear information, and then subsequently decide whether to respond. Therefore, reduced responses lead to better performance in organizations that have a high strategic orientation toward action.

### Action complexity

Considering the aspect of information processing and analysis mechanisms, information about external circumstances must be analyzed and transferred to the decision-maker. However, if an organization does not have a specific system in place or a return path to the administrator for a response, the organization’s response speed could decrease or be delayed [11]. In other words, the defender requires more information to support decision-making when they are unsure of the actual type of competition or the manner in which the initiator will defend their own benefit [10]. Therefore, when organizations initiate several types of competitive actions, a more competitors are less able to respond, which delays the speed of their responses, and decreases their capability and the resources they have available to respond. This can significantly improve the firm’s performance [14, 15].

## Methods

### Sample

This study focuses on hospitals. Research on the industrial characteristics of hospitals occasionally focused on the subject of “competition” before the launch of the NHI. With the launch of the NHI and the start of the global budget system, hospitals felt competitive pressure. According to the Ministry of Health and Welfare (MHW), in August 2009, regional hospitals in central Taiwan with more than 250 general acute beds had 32.51 beds for every 10,000 individuals, which is higher than the average of 31.61 beds for regions around Taiwan. Thus, Chiang et al. [16] note that beds per capita figures can be viewed as proxy variables for competitive intensity. Therefore, regional hospitals in central Taiwan are more competitive. Between 2006 and 2008, the point value of the global budget for hospitals of central Taiwan was lower than the national average point value. The global budget payment system in Taiwan adopts an expenditure cap, which constrains rapid growth in costs. If the total amount claimed for reimbursement by a sector exceeds the preset ceiling, the point values (which determine the amount hospitals receive from the NHI per service provided) for that sector’s services could drop.

This reveals the competitive pressure faced by hospitals in central Taiwan, particularly in the case of medium- and large-scale hospitals. Thus, this study focuses on the medical centers and regional hospitals (20 hospitals) in central Taiwan.

Semi-structured questionnaires were used to investigate the possible actions of hospitals from March 2009 to May 2009. This included in-depth interviews with superintendents, deputy superintendents, and assistants of superintendents of 10 medium- and large-scale hospitals in central Taiwan. Thus, this study collected data on previous actions adopted by the hospitals, such as developing cosmetic medicine services, paid for privately by customers, participating in community health-prevention programs, establishing common laboratories among hospitals, and forming joint medical procurement teams.

In order to avoid the classification of repetitive or incomplete types of actions, senior supervisors of two regional hospitals and one medical scholar (who was familiar with hospital operations) were invited to test and verify the content of the items. The senior supervisors provided suggestions (twice) to enhance the expert validity of this study. Finally, 21 types of general actions were identified, including price cutting/promotion and service improvement/enhancement (see Table 3).

### Data collection

After reviewing previous studies on competitive dynamics, Smith et al. [17] suggested that data collection in research studies on actions and responses of high-technology (high-tech) firms from 1985 to 1986, and on competitive responses of computer retailers in 1988 were all based on questionnaires. In addition to the interviews conducted to classify the actions, this study considered 20 medium- and large-scale hospitals, focusing on medical centers and regional hospitals in central Taiwan. Questionnaires were distributed to superintendents, deputy superintendents, and the supervisors responsible for the management of hospitals. The questionnaire included items of all competitive actions and perceptions of the hospital’s performance for the previous three years (i.e., 2006, 2007, and 2008).

### Measures

The definition and measurement of the independent variables are based on competitive dynamics theory. This study applies three independent variables as follows: implementation requirement of action [10, 6]; strategic orientation of action [11, 6, 13]; and action complexity [11, 10, 15, 14].

### Number of competitive action types adopted by hospitals

Observing a yearly pattern, regional hospital *G* has undertaken 20 types of actions, which is also the highest number of types of actions. Table 1 illustrates that medical center *C* has undertaken the most number of actions (346). Considering action numbers, medical center *C* and regional hospital *R* undertook the most actions for 2006 and 2007, and for 2008, respectively. Regional hospital *I* did not present any action type in 2006 because this was the year of its launch. However, the hospital began observable action types and numbers from 2007. Regional hospital *R* is one of the most interesting hospitals because it has grown in terms of both types of actions and the number of actions, as compared with other hospitals. Thus, is worth investigating further in future research.

**Table 1:** The types and number of competitive actions in hospitals

Names of hospitals	2006			2007			2008		
	Type of action^a^	Number of actions^b^	Objective performance^c^	Type of action	Number of actions	Objective performance	Type of action	Number of actions	Objective performance
Medical center A	17	96	6266	17	96	6314	17	100	6606
Medical center B	18	106	6656	18	115	7014	18	114	8096
Medical center C	19	116	3308	18	113	3512	18	117	3574
Medical center D	16	75	6606	16	77	6745	18	93	6977
Regional hospital E	16	75	1025	17	85	1110	18	91	1232
Regional hospital F	14	97	3141	15	94	3197	16	99	3215
Regional hospital G	20	102	1410	20	110	1364	20	109	1395
Regional hospital H	18	90	1617	18	94	1642	19	96	1657
Regional hospital I^d^	0	0	0	12	50	720	10	41	1107
Regional hospital J	16	92	3251	17	98	3383	18	107	3403
Regional hospital K	19	81	2831	19	100	2932	19	111	2951
Regional hospital L	13	56	1574	14	62	1591	15	65	1688
Regional hospital M	12	47	555	13	49	554	14	57	592
Regional hospital N	16	69	989	17	98	1011	18	108	1013
Regional hospital O	15	58	979	17	70	1219	18	78	1341
Regional hospital P	17	82	2824	17	82	2773	18	84	3059
Regional hospital Q	14	53	637	14	58	621	16	68	634
Regional hospital R	13	57	302	15	103	1064	19	133	1424
Regional hospital S	15	49	726	16	60	752	20	82	810
Regional hospital T	11	36	535	15	53	540	15	56	567
Total of year	–	1437		–	1667		–	1809	

^a^The type of action meaning may be influenced by the competitive impact and the attack intensity of the action

^b^The number of action means the action accumulation amount

^c^Objective performance means medical expenditures (i.e., outpatient service, hospitalization, dialysis, emergency treatment, etc.) from the NHI, which are applied by the hospitals every year on the NHIA website, and the unit is million points

^d^This hospital was founded in December 2006 and thus, type of action and the number of action in the said year are 0

#### Competitive actions

Competitive actions refer to specific and detectable competitive moves [11], such as the procurement of high-tech equipment. The questionnaire lists possible items from 21 types of actions. The total number of competitive actions of the hospitals in each year are based on the number of items in the current year, as indicated by the hospitals.

#### Implementation requirements of action

Based on the items in the questionnaire on action irreversibility designed by Chen and MacMillan [10], this study developed the “agreement with types of action” questionnaire, using a 5-point Likert scale (from “strongly disagree” to “strongly agree”), where each type included 12 items. The average scores of the hospitals are based on the scores of agreement with the 21 types of actions in each hospital, divided by 21 (types of actions), and divided again by 12 (items of each type). The formula for determining the implementation requirements score is as follows: i/21/12 = $\sum \limits_{i=1}^{21}$.

#### Strategic orientation of action

According to the results of the “agreement with types of actions” questionnaire, this study calculates the overall average of the hospital and the average of the execution conditions as 3.5694. When the score of the type of action is higher than the average of the overall implementation requirements, this is considered a strategically competitive action; when it is lower than the average, this is regarded as a tactically competitive action. Here, 11 types of actions revealed scores higher than the overall average of implementation requirements (strategic competitive actions). To measure the strategic orientation of actions, this study divided the total number of strategic competitive actions in the hospitals by the total number of their actions, and then multiplied this by the average of the overall implementation requirements.

#### Action complexity

This study multiplied the implementation requirement scores of the types of actions in one hospital by the total number of each actions in each year. A score higher than that of other hospitals means that the action had higher implementation requirements, and that the hospital was required to invest resources, which rivals struggled to compete with. The formula used was $\sum \limits_{i=1}^{21}$, where the implementation requirements score for type of action i * denotes the number of actions i.

#### Hospital performance

We include both objective and subjective performance in this study. Objective performance is measured in terms of annual medical expenditure (e.g., outpatient services, hospitalization, dialysis, emergency treatments, etc.) from the NHI, which hospitals apply every year based on the NHIA website [18]. Subjective performance refers to the perception and subjective feelings of senior managers toward their own hospital’s performance. However, hospitals’ performance cannot only be evaluated by medical expenditures of the NHI, and needs to include patients’ own expenditures. Similarly to previous studies [19–21], this study refers to the balanced scorecard [22] and the recommendations of professionals on hospital performance measurements. This study uses four dimensions of the balanced scorecard, including organizational learning and growth, internal processes, customer perspective, and financial dimensions. Finally, 10 items were included to enable each hospital’s senior supervisor to indicate the management team’s satisfaction with the yearly operation of the hospital on a 10-point scale (1 “strongly disagree”; 10 “strongly agree”). These items were medical quality, employees’ learning and growth, patients’ and relatives’ satisfaction, outpatient service personnel, emergency service personnel, hospitalization personnel, occupancy rate, income from customers’ own expenditures, use of high-tech medical equipment, and overall medical income for the year.

### Control variables

The critical control variables included in the study are ownership, evaluation level, and size of hospitals. From related research, ownership signifies that hospitals are divided into three categories, based on their ownership or profit share: public hospitals, private non-profit hospitals, and private for-profit hospitals [3, 4, 23–26]. Because ownership is a category variable, it is considered as a dummy variable. The evaluation level is based on the announcement of the Ministry of Health and Welfare. Similarly, the evaluation level of a hospital is also treated as a dummy variable. Organizational size is measured based on related research on hospitals [23, 24, 26]. Here, the variable is measured using the number of beds in a hospital, including acute beds, chronic beds, special beds, and observation beds. These organizational sizes are based on the number of beds registered on December 31, and the natural logarithms of these values are recorded for 2006, 2007, and 2008.

## Results

According to Table 2, among the implementation requirements of each type of action, “procurement of new medical equipment” and “enhancement of medical quality” have the highest implementation requirements points (3.9), followed by “mergers and acquisitions” (3.8625), and “development of featured medical service with characteristics” (3.8417). Among the 21 action types, 11 are higher than the mean 3.5694, and are thus referred to as “strategic actions.” Then, the other 10 types of actions are referred to as “tactical actions.” It can be observed from Table 2 that the identified strategic actions conform to the definitions of Chen et al. [6], who argue that strategic actions need greater resource commitments.

**Table 2:** Type of competitive actions of hospitals and the dimensions use to assess implementation requirements of action

Competitive actions	Implementation requirements of actions (mean)^a^	implementation requirements of actions dimensions
Procurement of new medical equipment	3.9000	● High capital should be invested when executing the action. ● The management should make significant efforts when executing the action. ● Personnel, system or process should be reconfigured when executing the action. ● The support of external stakeholders is required when executing the action. ● Senior supervisors should announce the execution of the action. ● The action should be significantly reported in the internal journals. ● Main stakeholders are the targets for responsibility and duty in the execution of the action. ● The equipment should be reorganized when executing the action. ● High level of cross-department integration is not required when executing the action. ● After the execution of the action, the levels of the organizations and authority should have high level of commitment. ● Senior supervisors should approve the execution of the action. ● Once the action is not executed, the related resources cannot be transferred to other actions.
Enhancement of medical quality	3.9000	
Merger and acquisition	3.8625	
Development of featured medical service	3.8417	
Development of innovative medical service	3.8250	
Expansion of service areas	3.7875	
Development of medical items upon customers’ expenditure	3.7333	
Service improvement/enhancement	3.6875	
Change of organizational structure	3.6292	
Participation in community service	3.6125	
Alliance	3.5917	
Acquisition of fair payment of medical cost	3.4958	
Share of financial responsibility	3.4792	
Different industry cooperation	3.4375	
Change of cost structure	3.4208	
Delegation affairs unrelated to NHI	3.3917	
Medical business outsourcing	3.3542	
Price cutting/promotion	3.3083	
Non-medical business outsourcing	3.2958	
Same industry cooperation	3.2125	
Bargaining of procurement	3.1917	

^a^Respondents rated each dimension on five-point scale (1, very low; 5, very high) for each competitive action. The index is mean

Table 3 presents the numbers of competitive action types over a three-year period. The most frequent competitive action types used by hospital managers are service improvement/enhancement (*n* = 796), followed by delegation affairs unrelated to the NHI (*n* = 480) and cooperation with other hospitals in the same industry (*n* = 456). The correlation coefficients are presented in Table 4.

**Table 3:** The number of competitive action type adopted by hospitals

The number of competitive action	Medical centers			Medical centers 3 years accumulation	Regional hospitals			Regional hospitals 3 years accumulation
Competitive actions type	2006	2007	2008		2006	2007	2008	
Procurement of new medical equipment	7	6	6	19	3	13	22	38
Enhancement of medical quality	21	21	22	64	63	72	71	206
Merger and acquisition	0	0	0	0	1	0	2	3
Development of featured medical service	17	17	21	55	41	48	61	150
Development of innovative medical service	30	32	38	100	51	87	100	238
Expansion of service areas	5	5	7	17	9	10	13	32
Development of medical items upon customers’ expenditure	36	37	40	113	78	92	105	275
Service improvement/enhancement	55	58	59	172	182	219	223	624
Change of organizational structure	5	5	5	15	14	14	16	44
Participation in community service	17	17	17	51	57	67	69	193
Alliance	0	0	0	0	30	33	36	99
Acquisition of fair payment of medical cost	20	20	20	60	58	65	70	193
Share of financial responsibility	7	7	6	20	19	19	21	59
Different industry cooperation	4	4	8	16	11	18	34	63
Change of cost structure	32	32	32	96	59	64	83	206
Delegation affairs unrelated to NHI	42	42	42	126	100	127	127	354
Medical business outsourcing	4	4	4	12	12	13	14	39
Price cutting/promotion	16	20	21	57	40	59	68	167
Non-medical business outsourcing	17	16	17	50	52	59	60	171
Same industry cooperation	38	38	38	114	101	119	122	342
Bargaining of procurement	20	20	21	61	63	68	68	199
Total	393	401	424	1218	1044	1266	1385	3695

**Table 4:** Pearson correlation analysis

Name of variables	1	2	3	4	5	6	7	8
1 Private non-profit hospital								
2 Private for-profit hospitals	−0.535**							
3 Regional hospital	−0.357**	0.327*						
4 Organizational size	−0.049	−0.050	−0.654**					
5 Implementation requirements	0.188	−0.220	−0.378**	0.489**				
6 Strategic orientation	−0.347**	0.248	−0.003	0.351**	0.295*			
7 Action complexity	−0.140	0.089	−0.386**	0.560**	0.497**	0.593**		
8 Perception performance	−0.420**	0.135	−0.101	0.351**	0.625**	0.592**	0.088	
9 Objective performance	0.041	0.097	−0.721**	0.803**	0.253	0.647**	0.242	0.377**

Number of samples (*n* = 20) is based on natural logarithm of organizational size and objective performance

***.Correlation is significant at the 0.01 level (2-tailed). **.Correlation is significant at the 0.05 level (2-tailed)

*.Correlation is significant at the 0.10 level (2-tailed)

### Results of regression analysis using generalized estimating equation models

Table 5 reveals the results of a hierarchical regression using generalized estimating equation (GEE) models. Model 1 is the result of the regression between the independent variables and subjective performance. Then, model 2 includes the control variables and independent variables. The result of the independent variables in relation to objective performance is presented in model 3. Lastly, the relationships between the control variables and independent variables, as well as their objective performance, are examined by including them in model 4.

**Table 5:** Hierarchical regression in Generalized Estimating Equation Models analytical result of hospital performance

Dependent variables: management performance												
	Model 1 interviewee perception performance			Model 2 interviewee perception performance			Model 3 Objective performance			Model 4 Objective performance		
Variables	B1	Std.Error1	Sig.	B2	Std.Error2	Sig.	B3	Std.Error3	Sig.	B4	Std.Error4	Sig.
Independent variables												
Implementation requirements	−20.281	13.9198		−20.920	15.8432		−0.121	0.2733		−0.417	0.1372	***
Strategic orientation	24.126	4.7518	***	20.745	5.9890	***	−0.145	0.2885		−0.009	0.2018	
Action complexity	0.086	0.0273	***	0.079	0.0272	***	0.003	0.0005	***	0.002	0.0004	***
Control variables												
Private non-profit hospital				−9.751	6.3849					0.043	0.0673	
Private for-profit hospitals				−7.451	5.9564					0.169	0.0757	**
Regional hospital				1.257	8.8418					−0.317	0.1262	**
Organizational size				0.005	0.0074					0.000	8.5350	***

Number of data (60 = 20 number of cluster*3 size of cluster), * *p* < 0.1; ** *p* < 0.05; *** *p* < 0.01

A hospital does not show better performance when it undertakes higher implementation requirements of actions. This means that higher implementation requirements result in a decrease in either the subjective (β = −20.920, *p*>0.1) or objective performance (β = −0.417, *p*<0.01) of hospitals. The strategic orientation of actions has a positive relationship with the perception of a hospital’s performance (β = 5.9890, *p*<0.01), but has no effect on objective performance (β = −0.009, *p*>0.1). Strategic actions are positively correlated with the perception of a hospital’s performance. Action complexity has a positive relationship with both subjective (β = 0.0272, *p*<0.01) and objective (β = 0.002, *p*<0.01) performance. Therefore, when hospitals adopt greater action complexity, this is expected to have a positive effect on both the subjective and objective performance.

## Discussion

### Implementation requirements of actions

Previous research argues that higher implementation requirements reduce the ability of competitors to respond and the speed of their responses [6]. Therefore, organizations undertake actions that result in a better performance. Actions require commitment from each level in the organization and certain implementation requirements, such as adjustments to the organizational structure and cooperation between teams. Certain actions, such as alliances or mergers and acquisitions, require more time and resources in order to integrate stakeholders and reorganize the organizational structure [6]. This makes it difficult to ascertain their effects on hospital performance, particularly over a short period. Here, no positive correlation is identified between subjective and objective performance when hospitals undertake actions with higher implementation requirements. Therefore, it is recommended that future research adopt a longitudinal design to follow the long-term effects of the implementation requirements of actions on hospitals.

### Strategic orientation of action

Among the actions undertaken by organizations, a greater strategic orientation results in fewer responses by competitors because organizations commit to their actions, thus deterring competitive responses [27]. Moreover, the efficiency of strategic actions could be uncertain, even after a relatively long period [28]. Therefore, competitors respond after the uncertainty disappears, which further motivates firms to undertake strategic actions.

Previous research has argued that strategic actions incur fewer and slower responses, resulting in a positive relationship between this kind of action and the benefits [6, 11]. Although the results of this study support the positive correlation between strategic action and the perception of a hospital’s performance, there is no positive correlation between these actions and objective performance.

The latter finding may be the result of the objective performance measurement, which uses the medical fees hospitals apply from the NHIA. However, some types of strategic actions, such as mergers and acquisitions, alliances, expansions of service areas, and the development of new medical services (e.g., micro cosmetic surgery, weight loss, etc.) should yield greater benefits to hospitals because they do not include an NHI payment or they are paid for by customers. Therefore, significant effects from objective performance cannot be observed.

### Action complexity

Several works argue that when organizations undertake more types of actions, they are more offensive than when using simple strategic actions, thus resulting in better performance [14, 15, 29]. Our results support that hospitals perform better as the action complexity increases. For example, myopia laser surgery, which many hospitals offer, differs in terms of patient satisfaction. Furthermore, many complex procedures, such as estimations before surgery, the stability of equipment, and the skills of surgeons entail long-term experience, customized surgery, and femtosecond non-knife cutting laser instruments. These factors make it difficult for hospitals to determine the results of these actions. Therefore, in order to perform better, competitors should not respond to the types of strategy actions that prompt hospitals to undertake these actions.

## Conclusions

Today, the global hospital management domain is highly competitive, and hospitals incur relative costs in response to competitive actions. Hospitals can undertake multiple actions when faced with environmental challenges [30]. This study provides hospitals with a concrete action type using competitive dynamics, offering management a different perspective from which to investigate their actions, as well as the implications of such actions. Hospitals should apply a competitive dynamic strategy to examine their own actions as well as those of their competitors. The healthcare industry should determine opportunities under the global budget policy by reducing medical payments under health insurance, given the medical regulations and generally compliant patients. Managers of hospitals should re-examine their strategic actions, and determine how other hospitals adopt appropriate strategies in order to survive in the competitive market.

### Limitations and suggestions for future research

We collected information on the action types and perceptions of hospitals’ performance for a three-year period using questionnaires. However, respondents do always clearly remember past actions in detail, which could cause a bias in the research results. In addition, although we included a category for “other” action types in the questionnaires, some actions may not be considered, or could be missed. Here, examples include items that belong to non-medical business outsourcing, such as waste disposal, electronic engineering, sludge disposal, public works, and air conditioning. In addition, the small sample size limits the generalizability to other fields.

Competitive interaction is a complicated and dynamic process [10]. Based on the results of the relationship between action characteristics and hospital performance, this study suggests that future research determine additional types of actions for each year and employ longitudinal data. Furthermore, future work should classify the 21 types of actions based on their customer orientation. In addition, the complex actions of hospitals change with the progress of technology. Lastly, researchers could collect data on the income generated by hospitals, including customers’ own expenses and registration fees, in order to determine why strategic orientation has no effect on objective performance.

## Abbreviations

AMC: Awareness-motivation-capability
MHW: Ministry of Health and Welfare
NHI: National Health Insurance
NHIA: National Health Insurance Administration
